# Bacteriophage purification using CIMmultus monolithic OH-column chromatography for therapeutic purposes

**DOI:** 10.1101/2025.02.03.636326

**Published:** 2025-02-03

**Authors:** Arne Echterhof, Tejas Dharmaraj, Patrick Blankenberg, Maryam Hajfathalian, Francis Blankenberg, Jessica Sacher, Paul L. Bollyky

**Affiliations:** 1 Division of Infectious Diseases and Geographic Medicine, Department of Medicine, Stanford University School of Medicine, Stanford, CA 94305, USA.; 2 Institute of Medical Microbiology, University Hospital of Muenster, Muenster, Germany

**Keywords:** Bacteriophage, Purification, FPLC, Chromatography, Endotoxin, CIMmultus Monolith

## Abstract

Bacteriophages, viruses that selectively infect bacteria, are increasingly explored as potential therapies to combat antibiotic-resistant infections. Effective phage purification is vital for therapeutic use, particularly to reduce endotoxin (lipopolysaccharide, LPS) and host protein contamination from production processes in gram-negative bacterial lysates. This study investigates the purification efficiency of CIMmultus OH-monolithic chromatography for seven similarly sized tailed phages (Myoviridae and Siphoviridae families) infecting *Pseudomonas aeruginosa*. Using preferential exclusion chromatography with a potassium phosphate gradient, we achieved significant reductions in host proteins and endotoxins, yielding therapeutically compliant samples suitable for human intravenous injection. We observed recovery rates of 85–95% for smaller phages (e.g., PAML-31–1, LPS5), with lower recoveries for larger “giant” phages (e.g., OMKO1, PhiKZ). All purified samples met endotoxin limits, with average reductions of 98.5% for proteins and up to 6.30 log reductions for endotoxins. Despite increased DNA content post-purification in most samples, the approach shows promise for clinical-grade phage purification. This chromatographic strategy is scalable, reproducible, and free from hazardous chemicals, making it suitable for industrial phage production.

## Introduction

Bacteriophages (Phages) are viruses that exclusively replicate in bacteria as their host organism. These nano-sized particles comprise proteins encapsulating a DNA or RNA genome [1]. Due to their selective antimicrobial targeting, bacteriophages are a potential treatment option for bacterial infection as an alternative or supplement to conventional antibiotics.

F́elix d’Herelle introduced phages as a novel antibacterial treatment and published non-randomized trials from medical experience over 100 years ago [2]. The dawn of the antibiotic age in the 1930s, the lack of reproducibility, and the contradictory results attenuate the enthusiasm for phage therapy in Western countries [3]. However, the emergence of antibiotic-resistant bacteria has restored researchers’ interest in reconsidering phages as a potential treatment for antibiotic-resistant pathogens [4,5]. To this day, there are no phage medications approved in Europe or the US hence phage therapy is applied under emergency investigational new drug (eIND) application or under the umbrella of Article 37 of the Declaration of Helsinki [6–8], nonetheless, clinical trials for various phage applications are ongoing [9,10].

Bacteriophages are the most numerous and ubiquitous biological entities on Earth. This is represented in the diversity of different phage morphologies which can be classified as tailed phages, tailless icosahedral phages, filamentous phages, and pleomorphic phages with or without lipid-containing envelopes. Besides diverse morphology, which leads to individual purification challenges among phage entities, phages must be produced in a bacterial host cell [11]. Consequently, key impurities might be host DNA host proteins, media components, and, in the case of gram-negative bacteria, Lipopolysaccharides (LPS). The fine line between removing key impurities while keeping the phages bioactive is the major hurdle in phage purification.

The downstream processing of the infected culture broth should isolate, concentrate, and purify the target phage. Usually, centrifugation and microfiltration steps are used to remove cells and cell debris [12]. Next, dead-end or tangential flow filtration, precipitation, gradient ultracentrifugation, or various chromatography approaches concentrate and purify phages. Phage purification for therapeutic applications requires additional standards since chemicals like CsCl, Triton 100, or chloroform commonly used for virus preparation are considered hazardous by regulatory agencies [11].

LPS remains critical in pharmaceutical production processes due to its ability to cause lethal immune responses in humans when intravenously injected at low-dose concentrations [13]. Lipopolysaccharides are molecules within the membrane of gram-negative bacteria and consist of three distinct regions: a hydrophobic lipid A region, a core oligosaccharide, and a hydrophilic O-specific polysaccharide [14]. As an endotoxin, it is released after cell death, which makes it abandonedly present in gram-negative bacterial lysate therefore, an endotoxin removal step must be conducted in every downstream processing step of gram-negative-based phage production. Due to their amphipathic properties, LPS molecules are difficult to remove from aqueous solutions based on their ability to form aggregates such as micelles (300–1000 kDa) and vesicles (>1000 kDa) stabilized by divalent Calcium and Magnesium cations [15]. The pyrogenicity of LPS requires removal in all biologicals, and the final concentration depends on its use. An intravenous or intrathecal application should not exceed the upper threshold of 5 Endotoxin Units (EU) daily per kilogram of body mass [16].

Phages are nano-sized particles and sensible to shear stress, which makes purification challenging in a conventional porous-solid matrices-based chromatography approach. Convective Interactive Media monolithic columns offer unique advantages regarding virus particle purification. The laminar flow created in monoliths reduces shear stress that leads to a higher recovery of big particles in comparison to bead or membrane-based columns independent of the flow rate, solute size, or viscosity. Additionally, the large channels reduce pressure drop across the column bed The surface of the highly interconnected network can be modified with different solid phase chemistries such as preferential exclusion columns, ion exchange columns, or hydrogen bond columns [17,18].

Preferential exclusion describes phenomena where co-solvents like cosmotropic ions are excluded from the solvation shell of solutes, here bacteriophages. In the presence of cosmotropic co-solvents like potassium phosphate, a water-cosolvent interaction is enthalpically favorable resulting in a salt-free area around biomolecules. A hydroxyl-substituted (OH) column is weakly hydrophobic and the solid phase is preferentially excluded, too. The biomolecules interact with the solid phase. Column retention of phages eliminates the target particle from the system by reducing the phage concentration from the solution. This reduces the overall salt-free area and, therefore, minimizes the free energy to reach a thermodynamically favored state. By reducing the salt concentration during the elution process, the particles are released from the solid phase and become soluble again [18–20].

CIMmultus monolithic columns have gained interest in the field of phage purification. Several papers describe using anion exchange monolithic chromatography to bind the negatively charged viral particle to the surface. Smrekar et al. used strong anion-exchange quaternary amine (QA) convective interaction media (CIM) monolith to capture a T4 phage from clarified E. coli lysate [21]. Adriaenssens et al. reported that eleven morphologically distinct phages can be purified by either QA or DEAE with a 40–99% recovery rate. However, neither paper includes measurements regarding LPS concentrations [22]. Van Belleghem et al. investigated the LPS removal from PNM and Luz 19 phages with an anion-exchange diethyl aminoethyl (DEAE) convective interaction media (CIM) monolith. They reported a limited reduction of endotoxin level and a strong titer reduction after chromatography [23]. Lee et al. demonstrated in their study that a CIMmultus monolithic OH column can be used in steric exclusion chromatography with a filamentous M13 phage by applying a polyethylene glycol (PEG) gradient. They report a 90% recovery and a reduction of 98% of host protein [17]. Rebula and colleagues present a two-step chromatographic purification approach for a PP-01 phage, including a capturing step with a CIMmultus monolithic OH column and a polishing step using three different column options CIMmultus with QA, H-Bond, and PrimaS ligands. They reported a 7-log endotoxin reduction by using H-Bond and PrimaS columns [18].

In our recent study, we investigated the potential of a 1ml CIMmultus monolithic OH column to purify seven similarly sized and shaped phages with a preferential exclusion approach using a potassium phosphate salt gradient.

## Results

### Phages with similar Morphology and DLS measurements can be purified using OH-monolithic chromatography

**Figure 1:**
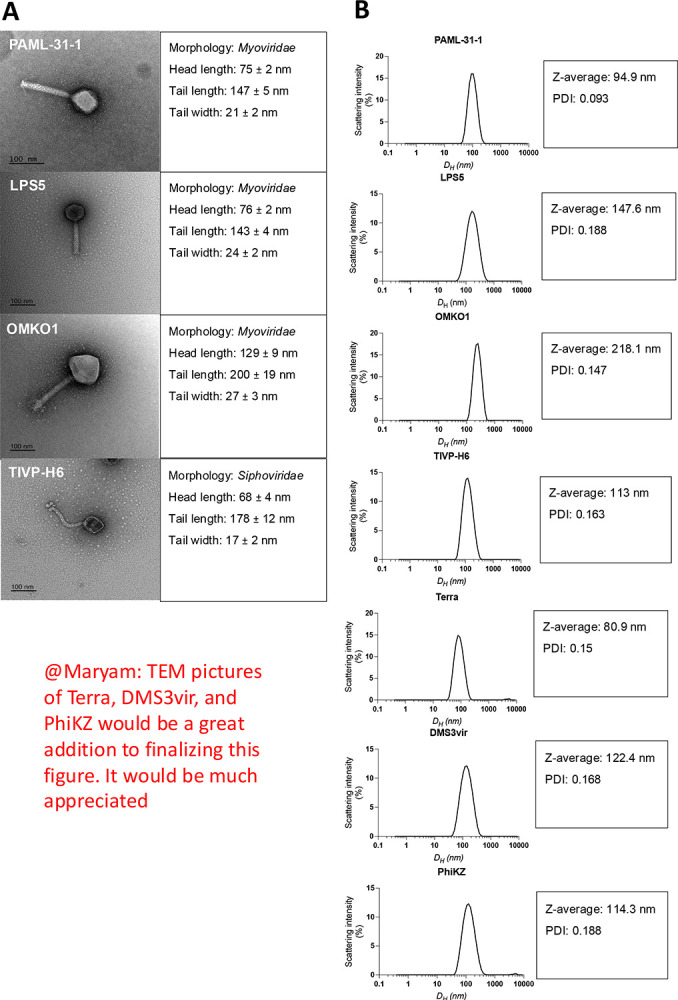
Size characteristics of 7 lytic *P. aeruginosa* phages used in this study. **A**. A representative transmission electron microscopy image of every is shown at 80,000x magnification. Head length, tail length, and tail width are measured on at least 5 different transmission electron microscopy examples for each phage**. B**. Dynamic light scattering (DLS) spectra, Z-average and PDI values of purified phages used in this study.

For this study, we investigated seven different *P. aeruginosa* phages regarding their purification potential using an FPLC chromatography system with a CIMmultus 1ml OH-monolithic column. All these seven phages share a similar-tailed phage-like morphology and similar dimensions. Our set of phages includes six members of Myvoridae PAML-31–1, LPS5, OMKO1, Terra, PhiKZ and DMS3vir (Fig 1). Within this group, OMKO1 And PhiKZ can be classified as giant phages. Furthermore TIVP-H6 belongs to the family of Siphoviridae. PAML-31–1 LPS5… share a similar head length from …nm to … nm and a tail length from … nm to … An exception regarding the tail length is TIVP-H6 with … nm. The two included Giant Phages PhiKZ and OMKO1 have a head length around … nm and a tail length of … nm. We report that those bigger phages have a lower recovery yield compared to the smaller phages tested in this study reaching from 42 to 35%.

The z-average measured by DLS does not correlate with the transmission electron microscopy size……

### OH-monolithic chromatography has a high recovery for phages of Myoviridae and Siphoviridae family

**Figure 2:**
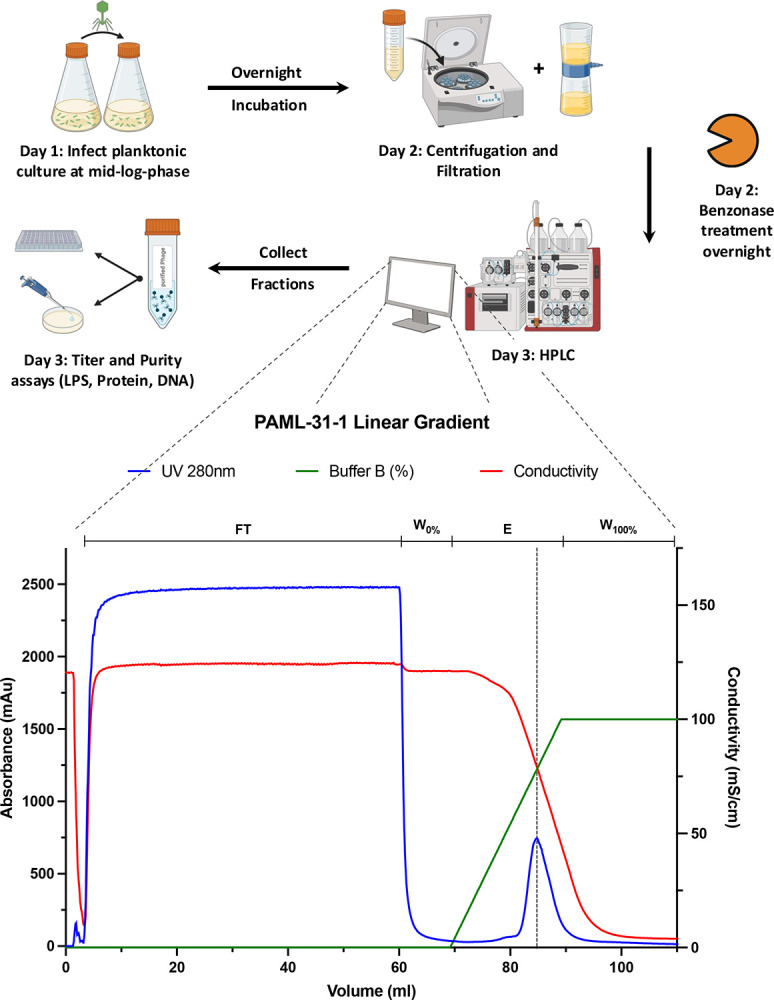
Monolithic Chromatography preparation of PAML-31–1 phage clarified lysate on CIMmultus OH 1ml column on Akta purifier HPLC system. 60 ml of PAML-31–1 phage lysate diluted to 1.5 M KH_2_PO_4_, pH 7.0 is loaded. Loading Buffer A: 1.5 M KH_2_PO_4_, pH 7.0, Elution Buffer B: 20mM KH_2_PO_4_, pH 7.0. Upper lines indicate different phases of the method: Flowthrough phase (**FT**), 10 CV washing the column after sample application with 10 CV (**W**_**0%**_), Elution with a linear Gradient over 20 CV to 100% Elution Buffer (**E**), final wash with 100% Buffer B to regenerate the column (**W**_**100%**_). Detection: blue: UV-absorbance (280 nm), red: Conductivity (mS/cm), green: Buffer B (%). Created in BioRender.com

The processed bacterial lysates of the seven lytic *P. aeruginosa* (PAML-31–1, OMKO1, LPS5, TIVP-H6, Terra, DMS3vir, and PhiKZ) phages were mixed with a 3M KH_2_PO_4_ buffer (pH 7.0) for a final salt concentration of 1.5M to match the salt potassium phosphate concentration of the binding buffer (buffer A). After column equilibration, the mixed phage samples were loaded into the FPLC system by setting the P960 sample pump to a flow rate of 1ml/min. The bound phages were eluted by linearly decreasing the potassium phosphate concentration to 20mM over 20 CV. Phage quantities of the sample application and the elution phase were assessed by plaque assay of the pooled collected fractions (Fig 2). Most of the phage input for each investigated phage sample was eluted in the collected elution fraction, while almost no phages were detected in the sample application phase. CIMmultus monolithic OH-column binds, concentrates, and elutes the seven investigated phages. The high UV absorbance in the sample application phase represents unbound impurities like host and media protein, whereas the sharp UV peak during the linear gradient represents the concentrated phage elution (Fig.2). The antipseudomonal phages PAML-31–1, LPS5 and TIVP-H6 showed the best recovery ranging from 85–95 percent. PAML-31–1 has a titer of 4.52E+11 PFU/ml in the elution fraction, and TIVP-H6 has a titer of 2.55E+11 PFU/ml. Terra and DMS3vir present with a 61% and a 41% recovery, reaching a final titer in the pooled elution of 1.14E+11 PFU/ml and a 9.00E+10 PFU/ml. Interestingly, the two giant phages included in this study, OMKO1 and PhiKZ indicate a lower recovery from the OH column ranging from 42–35%. OMKO1 has a titer of 2.14E+09 PFU/ml in the elution fraction, and PhiKZ has a titer of 1.01E+10 PFU/ml (Table 1). The lower recovery might be due to increasing shear stress over the sample application phase induced through already bound giant phages at the stationary phase. We observed an increased pressure escalation for OMKO1 over the sample application phase in comparison to the smaller phages like LPS5

### OH-monolithic chromatography effectively removes protein and endotoxin impurities from phage lysates

After removing the bacterial debris with centrifugation and a filtration step, the clarified bacterial lysates of all seven phages still contain protein and endotoxin impurities in abundance. The total protein content of all tested phage lysates is, on average, 1880 mg/ml, measured by a BCA assay. The total average lipopolysaccharide quantity of all tested phages lysates is 1.32E+05 EU/ml, measured with an Endozyme II assay. After performing a washing step of 10 CV to remove unbound samples from the stationary phase and after elution with a linear gradient over 20 CV, the endotoxin and protein were significantly removed. On average 98.5% of total protein was removed from the phage lysate. PAML-31–1 elution fraction contains 92.38 mg/ml total protein, which corresponds to a 1.34 log reduction, while LPS5 elution fraction holds 29.12 mg/ml of total protein, correlating to a 1.79 log reduction, and purified PhiKZ fraction comprises 17.39 mg/ml of total protein, which computes a 2.04 log reduction.

Our phage chromatography approach achieves an average endotoxins removal of 100% Terra elution fraction holds 66.62 EU/ml total LPS, correlating to a 2.89 log reduction, while LPS5 elution fraction contains 19.83 EU/ml of LPS, which corresponds to a 3.03 log reduction of LPS, and purified PAML-31–1 fraction holds 0.05 EU/ml of total LPS, which computes a 6.30 log reduction. For a summary of all purity parameters of all studied phages, see Table 1.

The preferential exclusion chromatography conducted with an OH-monolithic column using a potassium phosphate buffer regimen reliably purifies the seven tested Myoviridae and Siphovirus from protein and endotoxin contamination. All seven phages meet therapeutical standards for human intravenous injection regarding the endotoxin level with a given dose of at least 1E+10 PFU/ml per application applied three times daily for a hypothetical patient of 75kg.

We report a slight increase in DNA molecules after our preferential exclusion chromatography purification step. On average, the clarified and Benzonase-treated lysate comprises a total DNA concentration of 246.33 ng/ml. After purification, only the Terra elution fraction shows a decrease of DNA concentration of 85.83%, correlating with a 0.88 log reduction The other phage pooled elution fractions contain a higher DNA concentration than their corresponding pre-chromatography solution.

## Discussion

Here, we report a chromatographic bacteriophage purification approach using a CIMmultus 1 ml OH column in a preferential exclusion mode as a reliable, fast, and straightforward procedure to purify and concentrate seven similar-sized tailed bacteriophages (Myoviridae and Siphoviridae) against *P. aeruginosa*. Our purity assays reveal a significant LPS and protein reduction after FPLC for all the studied phages. The final LPS concentration of all phage samples meets therapeutical standards for intravenous injection, considering the maximum threshold of 5 Endotoxin Units (EU) daily per kilogram of body mass. The method can be conducted within three days without potentially hazardous substances like CsCl, chloroform, or Triton 100.

Convective Media Interaction (CIM) columns offer beneficial properties for purifying nano-sized particles like phages if binding and elution parameters are optimized. Fast and laminar flow rates combined with low shear stress, high capacities for large molecules, and flow-unaffected resolution create optimal phage purification conditions. Additionally, CIMmultus monolithic columns can be used multiple times and offer the option to scale up the purification production due to the availability of column volumes reaching from 1 ml to 8000 ml. When optimized, this chromatography approach can elevate bacteriophage purification to an industrial standard and would lower production costs if the equipment were regularly. Moreover, CIMmultus monolithic columns can be combined with versatile column chemistries such as anion-exchange columns like quaternary amine (QA), Diethyl aminoethyl (DEAE), Hydrogen Bond (H-Bond ADC) columns, multimodal anion exchange columns with hydrogen bonding potential (PrimaS) and weak hydrophobic hydroxyl-substituted (OH) column as it is used in this study. The adaptable array of column chemistry offers the potential to purify a broad spectrum of phages with diverse morphology. Further studies need to be conducted to optimize chromatographic parameters for individual phage structures.

Our chromatography approach increases the total DNA content in every Phage sample except Terra, although all phage lysates were incubated with Benzonase. These results are contradictory to Rebula et al. [18]. They report a 99% DNA reduction after a chromatographic step with a CIMmultus monolithic OH-column. It needs to be emphasized that this group does not treat the bacterial lysate with Benzonase. More investigations are needed to further understand the optimal way to reduce DNA content from phage lysates. This can be seen as one limitation of our study.

## Material and Methods

### Bacteriophage propagation and purification

Bacteriophages were propagated in a liquid culture in Erlenmeyer flasks at a suitable volume. LB medium was inoculated with the *P. aeruginosa* host strain and incubated until an early to mid-log growth phase was reached (37°C 275 rpm). Planktonic cultures were infected with a multiplicity of infection (MOI) of 0.01 and incubated overnight. Gross bacterial debris was removed by two to three cycles of centrifugation (8000xg, 20min 4°C) and the supernatant was filtered through a 0.22μm polyethersulfone (PES) membrane (Corning, NY, Product No. 4311188). The lysate was treated with 0.05U/ml Benzonase Nuclease (Sigma-Aldrich, St. Louis, MO, USA; Cat. No. E8263) overnight to digest free DNA.

### Monolithic chromatography phage purification

Benzonase-treated lysates were then mixed with 3M KH_2_PO_4_ buffer (pH 7.0) in a 1:1 ratio in preparation for purification by fast protein liquid chromatography (FPLC). The FPLC-based virus purification was conducted with a CIMmultus OH 1 mL monolithic column with a 2 μm channel size (Sartorius BIA Separations, Ajdovščina, Slovenia). The columns washing, regeneration, performance test, and storage were carried out according to manufacturer recommendations. The column was attached to an ÄKTA Pure FPLC (GE Healthcare Biosciences, Sweden) equipped with a P960 sample pump and analyzed with UNICORN 5.0 software (Cytiva Life Sciences, Marlborough, MA, USA). All purification protocols were run at room temperature and all buffers and samples were filtered through a 0.22 μm PES membrane. 10–60 mL of the mixed sample was loaded onto a pre-equilibrated column with 1.5 M KH_2_PO_4_ loading buffer, pH 7.0. After sample application, the column was washed with 10 column volumes of loading buffer to remove unbound particles. The elution of bacteriophage was performed using a linear gradient over 20 column volumes from 1.5M to 20 mM KH_2_PO_4_. The fraction corresponding to the eluted phage was collected based on UV A280 measurement. The column was cleaned by using a 1M NaOH solution followed by a pH neutralization step with 200mM Tris pH 7.5 and ddH_2_O, before Re-equilibration with 1.5 M KH_2_PO_4_ loading buffer, pH 7.0.

### Phage enumeration

Plaque assays were conducted to quantify the number of infectious phage particles per mL by using the spot dilution double agar overlay method. 100 μL of mid-log phase bacteria was added to 5 mL of top agar (5 g/L agar, 10 g/L tryptone, 10 g/L NaCl, 20 mM MgSO_4_, 20 mM CaCl_2_) and poured evenly onto the surface of an LB-agar plates to solidify. 10-fold serial dilutions of phages were prepared in 96-well polystyrene U-bottom tissue culture-treated plates (ThermoFisher Scientific, Waltham, MA, USA, Cat. No. 168136) in SM+gel buffer (50 mM Tris-HCl, 100 mM NaCl, 8 mM MgSO_4_, 0.01% gelatin (Sigma-Aldrich, St. Louis, MO, USA, Cat. No. G7041), pH 7.50). 10 μL of each dilution was spotted onto the top agar, incubated at 37 °C overnight, and visible plaques were counted to calculate the PFU/ml values.

### Total Protein, total DNA, and Endotoxin quantification

The total protein content of samples was measured using a bicinchoninic acid (BCA) assay (Thermo Scientific, USA, Pierce BCA Protein Assay kit Cat. No. 23225) according to the manufacturer’s instructions. The total DNA was determined by a Quant-iT PicoGreen assay (Invitrogen, USA Cat. No. P11496) according to the manufacturer’s manual. The endotoxin level of samples was measured using a recombinant Factor C Endotoxin Detection assay (bioMérieux, France, Endonext EndoZyme II assay Cat. No. 423129) with an assay range from 0.005 to 50 EU/ml. Samples were diluted with Endotoxin-free water to attain the assay range. All microplate assays were analyzed using an automated, multifunctional microplate reader (Tecan SPARK multimode reader, Switzerland).

### Transmission electron microscopy (TEM)

TEM imaging was done as previously reported [24]. In brief, the size and morphology of phages were examined with transmission electron microscopy (TEM) using a JEOL JEM1400 (JEOL USA Inc., Peabody, MA) at 80 kV. 5 μL of diluted phage solution was dropped onto carbon-coated copper grids (FCF-200-Cu, Electron Microscopy Sciences, Hatfield, PA). After 3 minutes, the grid was dipped into a ddH_2_O droplet and then 1% uranyl acetate for staining was dropped at the sample and was allowed to dry for 15 minutes before performing microscopy.

### Dynamic light scattering (DLS)

DLS measurement is well established in our laboratory [24]. Briefly, all DLS size distributions were conducted with a Nano-Series Zeta Sizer (Nano-NS ZEN3600; Malvern Instruments, Worcestershire, UK) equipped with a 633-nm laser. Measurements were obtained at 25°C at a backscattering angle of 173°. Each DLS measurement (single replicate) reported in this study is the average of 11 10-s measurements obtained after a 1-min vortexing period on half-speed, 1-min equilibration period in the instrument, and with variable attenuation to generate count rates >100 kcps. Phage diffusion coefficients were calculated from auto-correlated light intensity data, and hydrodynamic diameters (*D*H) were computed using ZetaSizer version 7 software with the Stokes-Einstein equation.

### Data availability

Data are available upon reasonable request from the corresponding authors subject to institutional review and approval. Supporting data values associated with the main manuscript is available in supplementary materials.

## Figures and Tables

**Table 1:** Summary of purification results. Titer (plaque assay), endotoxin (Endozyme II assay), protein (BCA assay), and DNA (Picogreen assay) concentrations before and after chromatography using the CIMmultus OH-column of six lytic Pseudomonas aeruginosa phages. Calculated values are phage recovery (%), impurity depletion (%), and impurity log reduction.

Phages	Titer			Endotoxin			
	(spot assay)			(Endozyme II)			
	Lysate (PFU/ml)	Elution (PFU/ml)	Recovery(%)	Lysate (EU/ml)	Elution (EU/ml)	depletion (%)	log reduction
**PAML-31–1**	1.85E+11	4.52E+11	0.94	1.09E+05	0.05	100.00	6.30
**OMKO1**	2.10E+09	2.14E+09	0.42	4.41E+04	0.47	100.00	4.97
**LPS5**	6.75E+10	3.10E+11	0.94	3.27E+04	19.83	99.99	3.22
**T4P-H6**	2.00E+11	2.55E+11	0.85	6.78E+04	35.51	99.96	3.28
**Terra**	1.25E+11	1.15E+11	0.61	5.15E+04	66.62	99.86	2.89
**DMS3vir**	1.48E+11	9.00E+10	0.41	4.54E+05	31.58	99.99	4.16
**PhiKZ**	1.93E+10	1.01E+10	0.35	1.85E+05	19.89	99.98	3.97

Phages	Protein				DNA			
	(BCA assay)				(Picogreen assay)			
	Lysate (mg/ml)	Elution (mg/ml)	depletion (%)	log reduction	Lysate (ng/ml)	Elution (ng/ml)	depletion (%)	log reduction
**PAML-31–1**	2031.69	92.38	98.14	1.34	461.14	1402.92		
**OMKO1**	1516.66	65.13	95.78	1.37	215.11	926.36		
**LPS5**	1792.98	29.12	99.65	1.79	277.52	856.66		
**T4P-H6**	1738.51	29.58	98.67	1.77	264.71	1155.81		
**Terra**	2031.00	17.12	99.08	2.07	123.08	16.04	85.83	0.88
**DMS3vir**	2139.04	27.33	97.91	1.89	190.91	226.99		
**PhiKZ**	1913.44	17.39	98.27	2.04	191.84	130.66		0.17
